# Neural Correlates of Orthographic Access in Mandarin Chinese Writing: An fMRI Study of the Word-Frequency Effect

**DOI:** 10.3389/fnbeh.2018.00288

**Published:** 2018-11-30

**Authors:** Yang Yang, Jun Zhang, Ze-Long Meng, Li Qin, Yu-Fei Liu, Hong-Yan Bi

**Affiliations:** ^1^Key Laboratory of Behavioral Science, Center for Brain Science and Learning Difficulties, Institute of Psychology, Chinese Academy of Sciences, Beijing, China; ^2^Department of Psychology, University of Chinese Academy of Sciences, Beijing, China; ^3^Center for Language and Brain, Shenzhen Institute of Neuroscience, Shenzhen, China; ^4^Jiangxi Institute of Education Sciences, Nanchang, China; ^5^School-family Partnership Research Center, Graduate School of Education, Peking University, Beijing, China

**Keywords:** Chinese writing, word-frequency effect, orthographic access, brain activation, functional connectivity

## Abstract

Writing is an essential tool for human communication and involves multiple linguistic, cognitive, and motor processes. Chinese, a logographic writing system, differs remarkably from the writing systems of alphabetic languages. The neural substrates of Chinese writing are largely unknown. Using functional magnetic resonance imaging (fMRI) in a copying task, this study probed the neural underpinnings of orthographic access during Mandarin Chinese writing by employing the word-frequency effect. The results showed that writing low-frequency characters evoked greater activation in the bilateral superior/middle/inferior frontal gyrus, superior/inferior parietal lobule, and fusiform gyrus than writing high-frequency characters. Moreover, psychophysiological interaction (PPI) analysis demonstrated that the word-frequency effect modulated functional connectivity within the frontal-occipital networks and the parietal-occipital networks. Together, these findings illustrate the neural correlates of orthographic access for Mandarin Chinese writing, shedding new light on the cognitive architecture of writing across various writing systems.

## Introduction

Written production is an important form of human language involving multiple cognitive, linguistic, and perceptual-motor operations that can be broadly divided into two components: central and peripheral processes. The former refers to the processing involved in the retrieval of orthographic codes, and the latter refers to the motor processing. Compared with other forms of language processing (e.g., speech production and reading), our knowledge of written production and particularly its neural substrates is relatively limited.

The cognitive processes and corresponding brain substrates of orthographic access during writing are critical open questions. A large body of lesion studies provide value clues to these questions by reporting different types of agraphia that selectively affect one set of mechanisms while sparing others. For example, lexical agraphia is manifested as selective impairments in spelling orthographically irregular and ambiguous words, along with a relatively intact ability to spell regular words and non-words (Beauvois and Dérouesné, 1981; Rapcsak and Beeson, 2004); this form of agraphia is thought to consist of damage to the whole-word retrieval process. Lesions of the angular gyrus (Anderson et al., 1990), the posterior inferior temporal gyrus (fusiform gyrus) (Rapcsak and Beeson, 2004), and the inferior frontal gyrus (Hillis et al., 2004) have been demonstrated to be associated with lexical agraphia. Phonological agraphia is another type of writing impairment characterized by specific difficulties in writing pronounceable non-words as well as unfamiliar or novel real words, with a well-preserved ability to write orthographically irregular words; this condition is attributed to the impairment of phoneme-grapheme transformation at the lexical or sublexical level (Shallice, 1981; Roeltgen and Heilman, 1984; Anderson et al., 1990). Damages to several brain regions including the supramarginal gyrus (Anderson et al., 1990), the posterior middle frontal gyrus (Sakurai et al., 1997), the posterior inferior frontal gyrus (Philipose et al., 2007), and the superior/middle temporal gyrus (Roeltgen and Heilman, 1984) has been proposed to be related to phonological agraphia. Furthermore, cases of pure agraphia featuring specific impairment of written output with normal reading skill (Beauvois and Dérouesné, 1981; Roeltgen and Heilman, 1984; Rapcsak et al., 1988) or oral spelling skill (Friedman and Alexander, 1989) suggest that orthographic access for written production involves specific processing mechanisms.

Neuroimaging studies have explored the neural basis of orthographic processing for written production in neurologically intact individuals. Some studies have used spelling judgment tasks in which participants were required to press buttons to report the correct spelling of words or letters presented visually or auditorily. Several brain regions have been found to subserve the retrieval of orthographic codes, including the left posterior inferior frontal gyrus (Rapp and Lipka, 2011; Purcell et al., 2017), the fusiform gyrus (Kronbichler et al., 2004; Philipose et al., 2007; Rapp and Dufor, 2011), the supplementary motor area (SMA) (Purcell et al., 2017), and the superior parietal lobule/inferior parietal sulcus (Purcell et al., 2011). However, such spelling judgment tasks are inherently different from actual writing. Thus, another line of research has examined the neural basis of orthographic access for written production after controlling motor components during actual writing tasks; such studies have demonstrated that the left inferior temporal gyrus (fusiform gyrus), the left inferior frontal gyrus, the middle temporal gyrus, and the inferior parietal lobule are consistently recruited to support the central component of writing (orthographic access) (Rapp and Dufor, 2011; Planton et al., 2013). By manipulating word-frequency and word-length effects in a spelling-to-dictation task, an fMRI study found that the left inferior frontal gyrus and the mid-fusiform gyrus were sensitive to word frequency, while the left superior frontal sulcus and the left superior parietal lobule was sensitive to word length (Rapp and Dufor, 2011). These results suggest that the fusiform gyrus and the inferior frontal junction are the neural loci of orthographic long-term memory, while the superior frontal sulcus and the superior parietal lobule subserve the orthographic working memory function. Together, previous studies have extablished that the fusiform gyrus/ventral occipitotemporal cortex and the superior and inferior frontal gyrus engage in orthographic access in written production. Furthermore, the ventral occipitotemporal cortex (vOTC) was found to be involved in both reading and writing processes, suggesting that writing calls upon a shared orthographic representation for writing and reading (Purcell et al., 2017).

Interestingly, evidence from lesion studies demonstrated the dissociation of writing impairment between kanji (a logographic writing system) and kana (an alphabetic writing system) (Tanaka et al., 1987; Sakurai et al., 1997), suggesting that the neural pathways involved in writing may vary across different writing systems. Chinese is a unique language system that differs dramatically from alphabetic languages. The basic orthographic unit of written Chinese is the character, a combination of radicals that are formed by strokes. In contrast to the linear structure of alphabetic words, which are constructed from sequences of letters, Chinese characters have a square configuration, presenting a high level of visual complexity. In Chinese, one Chinese character corresponds to a syllable, but there is no one-to-one correspondence for grapheme-to-phoneme conversion (GPC). Moreover, numerous homophones exist in Chinese with their own orthographic forms can share a single syllable, leading to arbitrary orthography-phonology correspondences in Chinese. Previous neuroimaging studies have demonstrated the specificity of neural substrates of Chinese reading (Siok et al., 2004, 2008; Tan et al., 2005a; Ge et al., 2015). For example, the left middle frontal gyrus and the right fusiform gyrus/occipital cortex have been found to be specific to orthography-to-phonology transformation (Tan et al., 2005a) and visual-spatial analysis (Wu et al., 2012), respectively. Accordingly, it is plausible that the features of Chinese characters would yield unique signatures of brain substrates associated with Chinese writing.

Some efforts have been made to unveil the neural basis of Chinese writing. For example, Cao and Perfetti (2016) directly compared the brain activation associated with mental writing of Mandarin Chinese characters and English words in a group of native speakers of Chinese and a group of English-speaking learners of Chinese. In that study, Chinese speakers showed greater activation in the middle frontal gyrus than English speakers, favoring the hypothesis that the neural substrates of written production vary across language systems (Tan et al., 2001, 2005a). More recently, another fMRI study examined the word-frequency effect during writing and reading of traditional Chinese characters. The word-frequency effect was detected in reading and in writing, involving the left middle occipital gyrus, the inferior occipital gyrus, and the left fusiform gyrus (Chen et al., 2016), which contrasts with the findings for alphabetic languages (Rapp and Dufor, 2011). The discrepancy may be derived from differences between the task paradigms. Chen et al. (2016) used a mental writing task, but Rapp and Dufor (2011) employed an actual writing task. Mental writing is inherently different from the process involved in actual writing, and their brain substrates differ as well (Planton et al., 2013). Furthermore, the behavioral responses involved in writing cannot be monitored during mental writing. As a result, the neural correlates of Chinese writing need to be investigated in an actual writing situation.

Using fMRI, the present study examined the neural underpinnings of orthographic access during actual writing of Mandarin Chinese characters. Following previous studies of reading (Kuo et al., 2003) and writing (Zhang and Wang, 2014; Qu et al., 2016), we employed the word-frequency effect as a probe to examine orthographic access during writing. High-frequency characters have a lower threshold of activation than low-frequency characters and are thus less vulnerable to impaired access. Consequently, the contrast between high- and low-frequency characters would be sensitive to the neural correlates of orthographic access during the writing of Chinese characters. We hypothesized that the word-frequency effect would be observed in the brain networks of visual-orthographic representation in the bilateral inferior temporal gyrus (fusiform gyrus), as well as the brain regions that support the orthographic retrieval process including the inferior frontal gyrus, the inferior parietal lobule and the motor regions. Moreover, we employed functional connectivity analysis to explore how the distinct brain regions interact in response to the word-frequency effect, which would be beneficial for delineating a full map of the neural substrates required for orthographic access in Chinese writing.

## Methods

### Participants

Thirty-four participants were recruited to participate in this study (18 male and 16 female; mean age =22.44 years, range 19 to 28 years). The mean education time of the participants was 15.74 years [standard deviation (*SD*) = 1.71]. The participants were undergraduate and graduate students who were recruited through advertisements posted on internet communities. The inclusion criteria were as follows: (1) Chinese as a native language; (2) age ranging from 18 to 30 years (3) right-handedness as assessed by a handedness inventory (Snyder and Harris, 1993); and (4) normal or corrected-to-normal vision. The exclusion criteria were as follows: (1) physical problems; (2) a history of neurological disease or psychiatric disorders; (3) other contraindications to safe MRI scanning, including metal fragments or implants. The study was approved by the ethics committee of the Institute of Psychology, Chinese Academy of Sciences, and the precedures were carried out in accordance with the approved guidelines. Prior to the experiment, written informed consent was obtained from each participant in accordance with the Declaration of Helsinki.

### Stimuli and task procedure

In the present study, we used a delayed copying task in which participants were required to copy Mandarin Chinese characters after that had been presented visually. Thirty characters were selected; and half of the characters were of high frequency **(**more than 50 occurrences per million) and the other half were of low frequency (no more than 1 occurrence per million) (Tan et al., 1995). The mean frequency of the high-frequency characters was 950 times per 1 million (*SD* = 327), and for low frequency character was 0.9 time per million Supplementary Material Table 1, according to the Modern Chinese Frequency Dictionary (1986). To control the variability of motor execution, we matched the mean number of strokes between high-frequency and low-frequency characters(3.31 for high-frequency characters and 3.35 for low-frequency characters; *t* = −1, *p* = 0.334). In addition, we employed a task in which participants were ask to copy geometric symbols to control for the activation elicited by motor and basic visual processing. Participants were instructed to use their normal writing style while minimizing the movements of their upper arm and forearm, and to match the duration and size between writing characters and drawing symbols.

**Figure 1:**
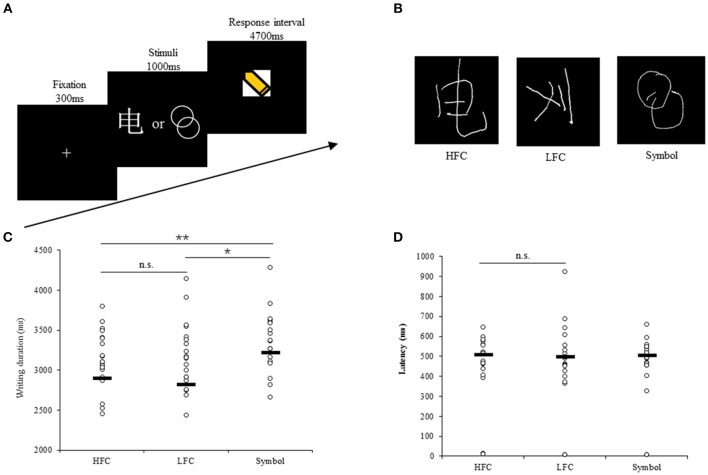
Experimental design and behavioral recordings during fMRI scanning. **(A)** Temporal structure of each task trial. **(B)** Examples of behavioral response of writing characters and drawing geometric symbols. Scatter plots of writing duration **(C)** and latency **(D)** in each task. The black lines show the group median. n.s., not significant. *p < 0.05, **p < 0.01.

A block design was employed, consisting of six blocks of copying characters (three blocks each for high-frequency and low-frequency characters) and three blocks of drawing symbols, in a pseudorandom order. Each block included visual presentation of instructions for 2 s followed by five trials. In each trial, a “+” symbol was first presented visually and centrally for 0.3 s, followed by presentation a stimulus for 1 s; next came a response period of 4.7 s with presentation of the writing cue of yellow pencil (Figure 1A). Four blocks of central fixation each with a 12-s duration were also interspersed among the task blocks as a “rest” condition. The total time for this design was 318 s.

Handwriting data were recorded using a tablet system specially developed for use in fMRI experiments. The tablet system includes a touch-sensitive surface, a force-sensitive stylus and an adjustable support frame, and is MRI-safe without significantly degrading fMRI data quality (Tam et al., 2011). The support frame was adjusted carefully for each participant to ensure that writing and drawing could be undertaken comfortably throughout the imaging session, and to enable tablet interaction with the forearm or wrist resting on the support such that there was no fatigue from writing against gravity (Figure 1B). The stimuli presentation was controlled by the E-Prime 2.0 software package.

#### Imaging acquisition

MRI data were continuously collected on a Siemens Prisma^fit^ 3T MRI scanner using a standard 20-channel coil at the Beijing MRI Center for Brain Research of the Chinese Academy of Sciences. Functional MRI time series data were acquired using a multiband BOLD-sensitive T2^*^-weighted gradient echo-planar-imaging (EPI) sequence (Moeller et al., 2010) (TR = 1,000 ms, TE = 30 ms, slices thickness = 2.2 mm, in-plane resolution = 2.2 × 2.2 mm, flip angle = 45°, phase encoding direction = AP, multiband acceleration factor = 4). Sixty-four axial slices covering the whole brain were collected using interleaved multislice mode in the ascending direction. Six “dummy” scans were acquired and then discarded by the scanners. High-spatial-resolution anatomical images were acquired using a T1-weighted, magnetization-prepared rapid acquisition gradient echo (MPRAGE) sequence (TR = 2,200 ms, TE = 2.08 ms, slice thickness = 1 mm, in-plane resolution = 1.0 × 1.0 mm and flip angle = 8°). Fat suppression was applied for T1 acquisition.

### Data analysis

#### Behavioral data

For the behavioral data, we collected data on writing duration and writing latency. The writing duration was defined by the interval from the start of the response period (first contact with the tablet) to the end of the last stroke of the response, while the writing latency was defined by the time period between the onset of the response screen and the first contact with the tablet. A one-way ANOVA was conducted to examine the differences in writing duration and latency between stimulus types (high-frequency characters vs. low-frequency characters vs. geometric symbols). In addition, the correlation between these two behavioral indexes in each condition was examined.

#### fMRI data

##### Preprocessing

Image preprocessing and statistical analyses were conducted by using SPM8 (http://www.fil.ion.ucl.ac.uk/spm/, Wellcome Department of Cognitive Neurology, University College London, London). The fMRI time series data for each participant were first corrected for head motion with reference to the first scan, and the realigned images were coregistered to the high-resolution T1 anatomical images using normalized mutual information. The anatomical images were normalized to Montreal Neurological Institute (MNI) stereotactic space via the unified segmentation approach, and the resulting warp parameters were applied to the normalization of functional images onto the MNI space with cubic voxels at a spatial resolution of 2 × 2 × 2 mm. The normalized functional images were then smoothed with an isotropic Gaussian kernel with 6 mm full-width at half-maximum. One participant was excluded from subsequent analysis due to excessive head motion (>2.5 mm translation or > 2.5° rotation). Another participant was excluded due to signal loss in the prefrontal cortex.

##### Brain activation analysis

At the first level, activation maps contrasting copying characters to drawing geometric symbols were generated for each participant by using the general linear model (GLM) method. The GLM design matrix included the block design time series of writing and drawing tasks convolved with a canonical hemodynamic response function. To minimize residual motion artifacts, we included head movement parameters (estimated with six degrees of freedom during the motion correction step) in the design matrix as nuisance covariates. The data were processed with a high-pass-filtered at 128 s. An autoregressive model of order one (AR (1)) was applied to correct the residual intrasubject temporal correlation. For each participant, brain activation was first assessed for four contrasts (high-frequency characters > geometric symbols, low-frequency characters > geometric symbols, high-frequency > low-frequency characters and low-frequency> high-frequency characters). At the second level, the individual activation maps were entered into a second-level random-effects model using a one-sample *t*-test. To accommodate the influence of motor differences between writing characters and drawing symbols (see the Results section), we included the differences in writing duration (drawing symbols vs. low-frequency or high-frequency characters) as covariates for the activation analysis of high- and low-frequency character conditions. The voxelwise threshold for statistically significant activation was set at *p* < 0.05, false discovery rate (FDR) corrected for multiple comparisons with a minimum cluster extent of 20 contiguous voxels. The locations of the brain regions were estimated from the Talairach atlas (Talairach and Tournoux, 1988).

##### Functional connectivity analysis

Psychophysiological interaction (PPI) analysis implemented in SPM8 was applied to compute the functional connectivity associated with the word-frequency effect during Mandarin Chinese writing. PPI analysis illustrates task-dependent interaction between an a priori defined seed region and all voxels in the rest of the brain based on multiple regression models (Friston et al., 1997). In this study, functional regions of interest (ROIs) were defined based on the results of group analysis of the frequency effect (low-frequency > high-frequency characters), and all the ROIs were constrained within the networks for which the word-frequency effect has been identified in reading or spelling processes (Kuo et al., 2003, 2004; Rapp and Dufor, 2011; Rapp and Lipka, 2011). Accordingly, several ROIs were selected including the bilateral superior frontal gyrus (x = −6, y = 12, z = 51; right: x = 4, y = 16, z = 49 in Talairach coordinates), the posterior inferior frontal gyrus (left: x = −44, y = 11, z = 29; right: x = 48, y = 11, z = 25), the inferior parietal lobule (left: x = −38, y = −37, z = 39; right: x = 30, y = −42, z = 48), and the fusiform gyrus (left: x = −40, y = −45, z = −15; right: x = 44, y = −51, z = −8). ROIs were created as spheres with a radius of 8 mm. For each ROI, a regression model was built including three regressors: PPI, the physiological regressor of the ROI (eigenvariate) and the psychological regressor (word-frequency effect: low-frequency > high-frequency characters). The activation maps of PPI for each ROI and for each participant were entered into a second-level group analysis using a one-sample *t* test. The threshold was set at *p* < 0.001 uncorrected at the voxelwise level and *p* < 0.05 with FDR correction at the cluster level. Unthresholded statistical maps of the main results have been unloaded to the NeuroVault repository (https://neurovault.org/images/100330/).

## Results

### Behavior

The behavioral data analysis was based on thirty-two participants, as two participants were excluded due to extreme head movement and signal loss in the frontal cortex, respectively. The average writing durations (*SD*) of high-frequency characters, low-frequency characters and geometric symbols were 2,838 ms (503), 2,878 ms (528), and 3,197 ms (437), respectively (Figure 1C). A one-way ANOVA analysis revealed a main effect of stimulus type [*F*_(2, 93)_ = 5.14, *p* = 0.008]. *Post-hoc* pairwise comparison showed that the duration of drawing geometric symbols was significantly longer than that of writing high-frequency characters (difference = 358 ms, *p* = 0.004) or low-frequency characters (difference = 318 ms, *p* = 0.011), but there was no difference between the high-frequency and low-frequency conditions (difference = 40 ms, *p* = 0.745).

The writing latencies (*SD*) of high-frequency characters, low-frequency characters and geometric symbols were 484 ms (143), 500 ms (185), and 475 ms (152), respectively (Figure 1D). Thirty participants were ultimately included in the one-way ANOVA analysis for latency because two participants whose responses were more than three SDs from the mean were excluded. No differences in latency between conditions were detected [*F*_(2, 91)_ = 0.6, *p* = 0.551]. Moreover, correlation analysis demonstrated that writing duration and latency were significantly correlated for high frequency characters (*r* = −0.34, *p* = 0.029), low frequency characters (*r* = −0.53, *p* = 0.002), and geometric symbols (*r* = −0.38, *p* = 0.034), consistent with the interaction viewpoint between central and peripheral processes during writing (Roux et al., 2013; Kandel and Perret, 2015; Zhang and Feng, 2017).

### Brain activation

**Figure 2:**
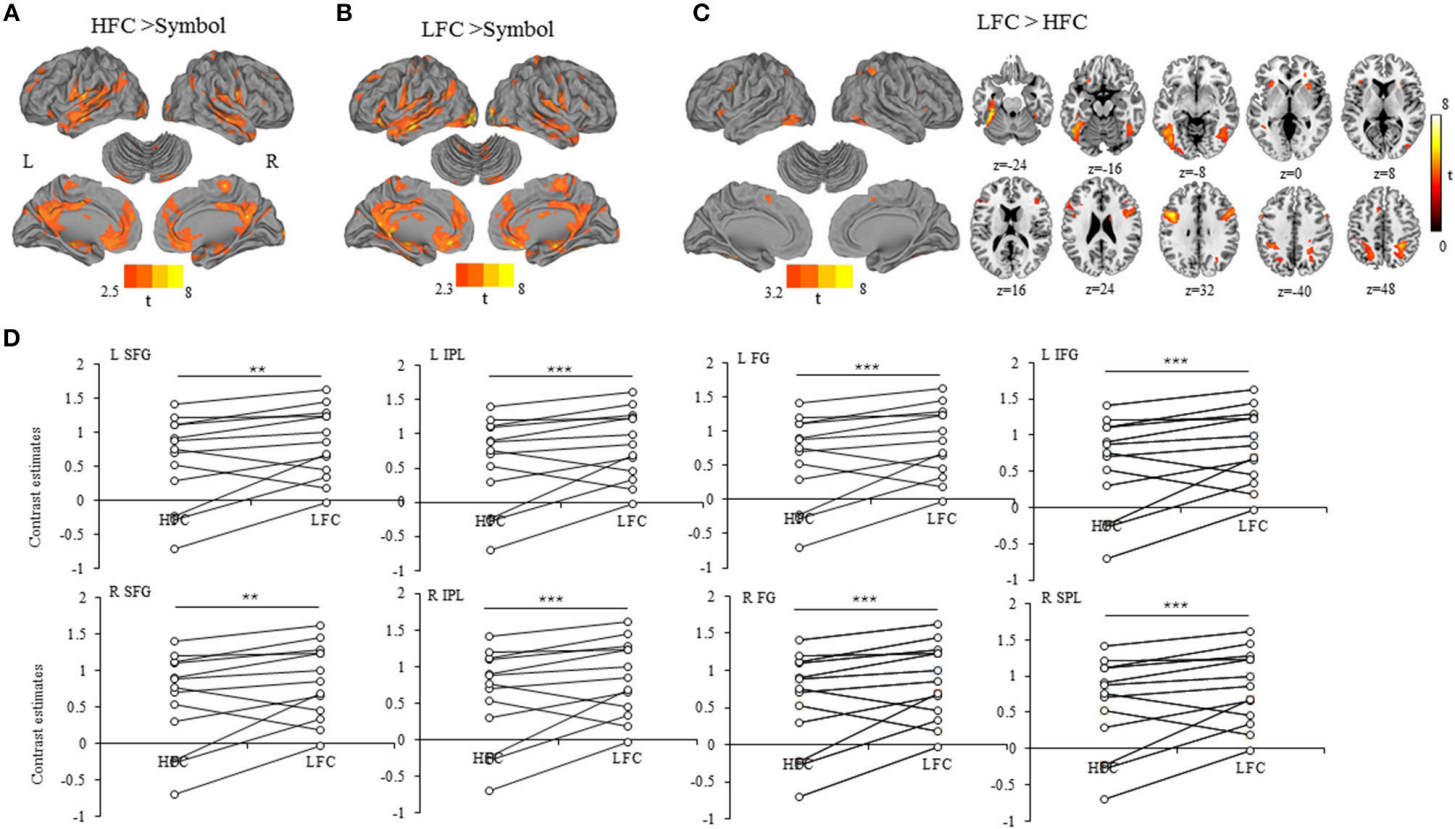
**(A)** Activation for the contrast writing HFC minus drawing symbols. **(B)** Activation for the contrast writing LFC minus drawing symbols. **(C)** Activation for the contrast writing HFC minus writing LFC. The activation clusters were visualized using the Caret software version 5.5 (https://www.nitrc.org/projects/caret/). **(D)** Scatter plots of contrast estimates of the brain regions in relation to the word-frequency effect (LFC > HFC). Each closed circle represents one participant, and the dark lines link the identical participants. HFC, high-frequency characters; LFC, low-frequency characters; SFG, superior frontal gyrus; IPL, inferior parietal lobule; FG, fusiform gyrus; IFG, inferior frontal gyrus; SPL, superior parietal lobule; L, left; R, right. **p < 0.01, ***p < 0.001.

We found that a large-scale neural network was recruited for copying either high-frequency (Figure 2A) or low-frequency Chinese characters (Figure 2B), involving the precentral gyrus, superior/medial/inferior frontal gyrus, cingulate gyrus, insula, superior/middle/inferior temporal gyrus, superior/inferior parietal lobule, middle/inferior occipital gyrus, caudate and cerebellum. Critically, the word-frequency effect was characterized by greater activation in the bilateral superior/middle/inferior frontal gyrus, bilateral superior /inferior parietal lobule (precuneus), middle occipital gyrus and bilateral fusiform gyrus for low-frequency characters than for high-frequency characters, while no significant activation was detected for the contrast between high-frequency and low-frequency characters (Table 1, Figure 2C). Contrast estimates of the brain regions identified by the contrast of writing low-frequency vs. high-frequency characters were shown in Figure 2D, which were extracted from spherical ROIs with 6-mm radius.

**Table 1:** Coordinates of activation peaks of the comparisons between writing HFC and writing LFC.

**Brain region**	**BA**	**x**	**y**	**z**	**z score**
R postcentral gyrus	3	28	−27	40	3.53
L superior frontal gyrus	6	−6	12	51	3.77
R superior frontal gyrus	8	4	16	49	3.7
R middle frontal gyrus	9	57	15	31	4.12
	11	22	25	−11	4.1
	46	42	28	19	3.49
L inferior frontal gyrus	47	−26	25	−15	3.98
	13	−40	28	10	3.87
	9	−44	11	29	5.45
	9	−55	21	25	3.72
	46	−53	30	17	3.41
R inferior frontal gyrus	9	48	11	25	4.92
	13	44	28	12	3.55
R superior parietal Lobulele	7	30	−56	47	3.75
L inferior parietal Lobule	40	−38	−37	39	3.98
				
	40	30	−42	48	4.72
L Precuneus	7	−20	−60	44	3.82
	7	−24	−58	51	3.79
R middle temporal gyruss	39	40	−77	13	3.32
R inferior temporal gyruss	20	51	−53	−9	4.34
L middle occipital gyrus	19	−50	−59	−5	4.81
L parahippocampal gyrusus	36	−34	−32	−19	5.21
R cingulate gyrus	31	24	−45	37	4.15
L claustrum		−30	23	−3	4.26
R claustrum		28	21	3	4.53
L fusiform gyrus	37	−40	−45	−15	5.51
R fusiform gyrus	37	44	−51	−8	4.06
	37	38	−43	−15	4.04
R caudate		12	1	20	3.75

*Z-score corresponds to the actual maximum pixel value within the brain region from the statistical parametric map. L,left; R, right; BA, Brodmann's area*.

### Functional connectivity

**Figure 3:**
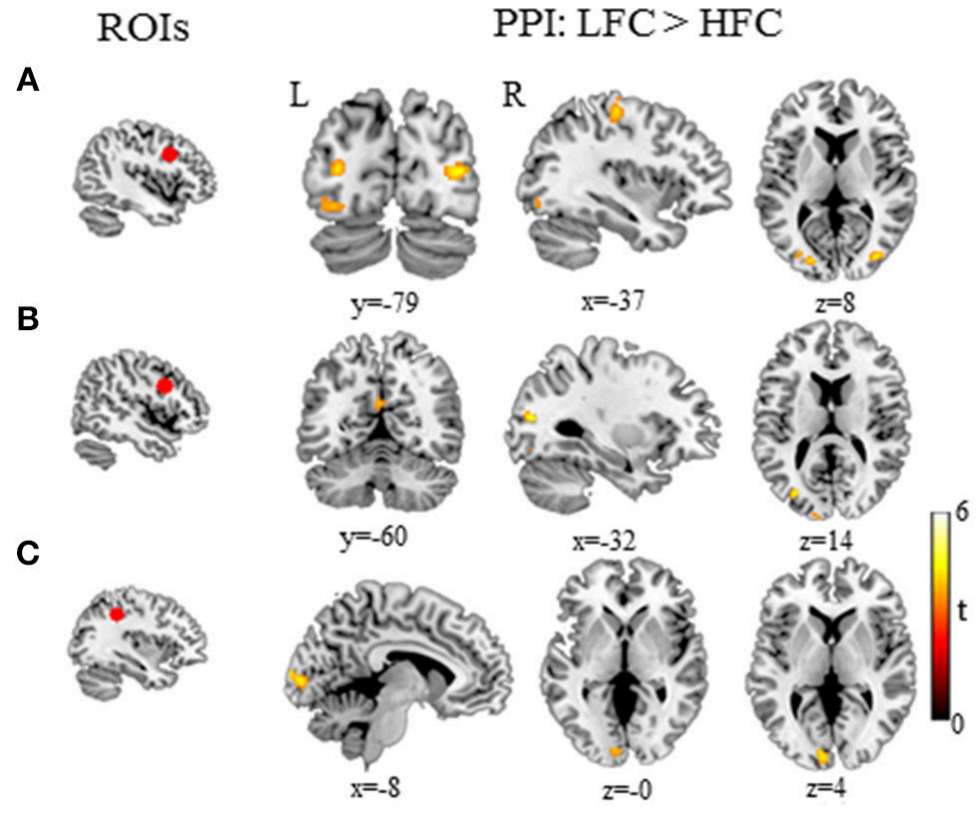
Functional connections associated with the word-frequency effect (LFC > HFC) during Chinese writing. **(A)** Brain regions that increased functional connectivity with the left right superior frontal gyrus for LFC relative to HFC. **(B)** Brain regions that increased functional connectivity with the left inferior frontal gyrus for LFC relative to HFC. **(C)** Brain regions that increased functional connectivity with the left inferior parietal lobule for LFC relative to HFC. HFC, high-frequency characters; LFC, low-frequency characters; L, left; R, right.

PPI analysis revealed a few functional networks modulated by the word-frequency effect (low-frequency>high-frequency characters) in Mandarin Chinese writing. First, we found that several neural pathways within the fronto-occipital circuits were sensitive to word frequency; these connections included those between the right superior frontal gyrus and the left precentral gyrus, the bilateral middle/inferior occipital gyrus (lingual gyrus) and the fusiform gyrus, and between the posterior inferior frontal gyrus and the left precentral gyrus, the middle occipital gyrus and the right inferior temporal gyrus. Second, we found that the word-frequency effect was related to the functional connectivity between the left parietal lobule and the left middle occipital gyrus and the cuneus (Table 2, Figure 3).

**Table 2:** Functional connectivity associated with the word-frequency effect (LFC > HFC) during Chinese writing.

**Brain regions**	**BA**	**x**	**y**	**z**	**z score**	**Voxels**
**R SUPERIOR FRONTAL GYRUS SEED**						
L precentral gyrus	6	−40	−13	60	3.75	121
	4	−40	−17	54	3.67
L middle occipital gyrus	19	−32	−81	17	5.26	2464
	18	−16	−91	16	3.71
	37	−40	−62	−2	3.95
L lingual gyrus	19	−30	−76	−5	3.73
R lingual gyrus	19	28	−60	−4	3.92
	18	4	−76	−6	3.91
R middle occipital gyrus	19	32	−81	13	4.6
	18	36	−85	3	3.7
L inferior occipital gyrus	19	−40	−76	−6	3.92	547
R inferior occipital gyrus	18	36	−82	1	3.74
L cuneus	17	−24	−85	15	4.58
L middle temporal gyrus	37	−46	−66	7	3.84
L fusiform gyrus	19	−32	−64	−7	3.32
R fusiform gyrus	37	50	−57	−11	3.95
**L INFERIOR FRONTAL GYRUS SEED**						
L precentral gyrus	6	−40	−16	60	3.34	100
L postcentral gyrus	3	−40	−19	53	3.75
L middle occipital gyrus	19	−32	−75	13	3.92	106
R middle occipital gyrus	19	34	−79	13	4.01	101
R inferior temporal gyrus	37	46	−68	−3	3.37
L cuneus	17	−22	−81	11	3.77
**L INFERIOR PARIETAL LOBULE SEED**						
L cuneus	17	−8	−91	8	3.88	115
L middle occipital gyrus	18	−6	−96	14	3.85

*Z-scores correspond to the actual maximum pixel value within the brain region from the statistical parametric map. L, left; R, right; BA, Brodmann's area*.

## Discussion

In the present study, we used the word-frequency effect to investigate the neural correlates of orthographic access in Mandarin Chinese writing. In terms of coupled brain activation and functional connectivity analysis, brain regions whose activation and interactions are necessary for the frequency effect were identified, involving the bilateral superior/middle/inferior frontal gyrus, superior/inferior parietal lobule, precuneus, and the fusiform gyrus, suggesting that these regions are the neural basis of orthographic access in Chinese writing.

### Brain activation for copying chinese characters

Unlike previous neuroimaging studies that used mental Chinese writing (Cao et al., 2013; Cao and Perfetti, 2016; Chen et al., 2016), the present study unveiled, for the first time, the brain mechanism of copying Chinese characters in the context of actual writing. Compared to drawing symbols, copying Chinese characters evoked brain activation in the bilateral precentral gyrus, the superior/middle/inferior frontal gyrus, the cingulate gyrus, the angular gyrus, the superior parietal lobule (precuneus), the superior/middle temporal gyrus, the fusiform gyrus, and the cerebellum, irrespective of word frequency. These regions have been implicated in central and peripheral processes in writing (Purcell et al., 2011a; Planton et al., 2013). Because low-level visual and motor processes were well controlled, the results of this study could be the specific brain substrates for Mandarin Chinese writing. In addition, previous behavioral studies have demonstrated that copying characters or symbols is uniquely essential for Chinese reading skill (Tan et al., 2005b; Guan et al., 2015; Kalindi et al., 2015); thus, our findings offer valuable information for understanding the relationship between copying skill and reading in Chinese.

### Brian activation associated with the word-frequency effect in the occipital/occipital-temporal cortex

Our examination of the word-frequency effect indicated that low-frequency characters evoked stronger activation than high-frequency characters in the bilateral mid-fusiform gyrus with peaks at Talairach coordinates of (−40, −45, −15) and (44, −51, −5), consistent with previous findings regarding writing or spelling processes in alphabetic writing systems (Rapp and Dufor, 2011; Rapp and Lipka, 2011). Activation in the left fusiform gyrus or ventral occipitotemporal cortex has been repeatedly reported in both reading and writing of alphabetic languages (Kronbichler et al., 2004; Rapp and Dufor, 2011; Rapp and Lipka, 2011; Planton et al., 2013; Schurz et al., 2014) and non-alphabetic languages (Nakamura et al., 2000; Tan et al., 2005a; Liu et al., 2008; Wu et al., 2012; Cao and Perfetti, 2016), despite variability in activation peaks. There are two prominent hypotheses concerning the role of the left mid-fusiform gyrus/ventral occipitotemporal cortex in visual word processing. One viewpoint is that this region is the specific neural substrate of orthographic representation of word in the long-term memory, known as the visual word form area (VWFA) (Dehaene et al., 2002; Dehaene and Cohen, 2011). Thus, the observed greater activation in the left fusiform gyrus for low-frequency than for high-frequency characters may occur because less effort is needed for the high-frequency characters, which are more readily matched with stored representations (Kronbichler et al., 2004). In contrast, other researchers have posited an interactive account of the role of the left fusiform gyrus/ventral occipitotemporal cortex in recognition of written words (Price and Devlin, 2011). Specifically, this regions has been proposed to be the interface linking low-level visual features and high-level language processing, such as phonology or meaning (Price and Devlin, 2011). Likewise, the increased activation for low-frequency characters in the left fusiform gyrus may reflect an increased demand for top-down modulation required during orthographic access to low-frequency characters for written production.

Furthermore, in contrast to the findings for alphabetic languages (Rapp and Dufor, 2011), we also found significant activation in the right fusiform gyrus for the word-frequency effect. This finding is compatible with previous neuroimaging studies of Chinese reading (Tan et al., 2001; Kuo et al., 2003; Liu et al., 2008; Wu et al., 2012; Xu et al., 2015). The complex visual-spatial attributes of Chinese characters may require additional processing for visual analysis, and thus the bilateral fusiform gyri should work together to support orthographic access to Chinese characters. The specific role of the right fusiform gyrus in Chinese processing is still elusive. One possibility is that this region subserves the integration of different radicals of Chinese characters (Liu and Perfetti, 2003). This explanation is in accordance with empirical evidence that radicals are the unit of written production in Chinese (Damian and Qu, Forthcoming). Together, this bilateral activation of the fusiform gyrus suggested that multiple-levels of visual-orthographic representation are engaged in Mandarin Chinese writing.

### Brian activation associated with the word-frequency effect in the frontal cortex

Two activation clusters in the frontal cortex were identified to be sensitive to the word-frequency effect. First, we found that low-frequency character yielded greater activation than high-frequency characters in the posterior inferior frontal gyrus extending to the middle frontal gyrus, with the peak at (−44, 11, 29), which was more anterior than the site identified in writing of alphabetic scripts (−49, 1, 33) (Rapp and Dufor, 2011). Our result is consistent with the finding of a frequency effect during Chinese reading (Kuo et al., 2003), supporting the hypothesis of shared orthographic processing between writing and reading (Rapp and Dufor, 2011; Purcell et al., 2017). Functionally, the dorsal posterior inferior frontal gyrus has been found to be associated with phonological processing in alphabetic languages (Poldrack et al., 1999; Booth et al., 2007; Cone et al., 2008) and Chinese (Tan et al., 2005a; Cao et al., 2011, 2017; Wu et al., 2012), including phoneme segmentation and manipulation (Booth et al., 2007) and integration of orthography, phonology, and semantics (Siok et al., 2004). For written production in particular, the posterior part of the inferior frontal gyrus has been reported to subserve phono-graphemic conversion in a writing-to-dictation task (Omura et al., 2004). Activation in the left inferior frontal gyrus might represent the phonological activation of characters during written production, in line with behavioral studies showing phonological interference for written naming (Qu et al., 2011). Alternatively, the posterior left inferior frontal gyrus has been suggested as the brain substrate for selection among orthographic forms (Badre et al., 2005; Moss et al., 2005); thus, the activation difference between low- and high-frequency characters may reflect a difference in selection demands among orthographic forms (Rapp and Dufor, 2011). Meanwhile, we found significant activation in the right inferior/middle frontal gyrus for the word-frequency effect. An fMRI study has suggested that the right middle frontal gyrus is the neural basis of orthographic processing in Chinese reading (Kuo et al., 2004). According to this view, the significant activation in the right inferior/middle frontal gyrus for the frequency effect may reflect the long-term representation or retrieval of orthographic forms of characters. Additionally, a well-known function of the right inferior frontal gyrus is inhibition and attentional control (Aron et al., 2003; Xue et al., 2008; Hampshire et al., 2010). The increased activation in the right inferior frontal gyrus for the low-frequency characters may be induced by the high demand for inhibitory control during written production due to the unfamiliarity of low-frequency characters.

The other cluster of significant activation in the frontal cortex was the superior frontal gyrus, corresponding to the pre-SMA, centered at (−6, 12, 51) and (4, 16, 49), which agrees with the finding of a frequency effect in Mandarin Chinese reading (Kuo et al., 2003; Carreiras et al., 2006). The SMA is a commonly reported region in writing studies, and has traditionally been thought to be a motor processing area (Planton et al., 2013). Functionally, this area is thought to be responsible for sequence processing (Crozier et al., 1999; Cona and Semenza, 2017), such as updating and switching of sequences (Bapi et al., 2006). Thus, the SMA might serve the dynamic representation of stroke sequence of Chinese characters, and the significant activation associated with the frequency effect might be derived from the low level of accessibility of writing sequences of the low-frequency characters. This viewpoint is consistent with previous observations that the SMA was significantly activated when subjects were shown the reverse writing sequence of Chinese characters (Yu et al., 2011).

### Brian activation associated with the word-frequency effect in the parietal cortex

Brain activation was also identified for the word-frequency effect in the parietal cortex covering the bilateral superior and inferior parietal lobule. The inferior parietal lobule has been claimed to subserve short-term phonological storage in alphabetical languages (Ravizza et al., 2004) and Chinese (Tan et al., 2005a). Based on the phonological mediation hypothesis (Geschwind, 1969), phonological activation is an obligatory route for orthographic access during writing and is utilized to support the one-to-one mapping between the phonology and orthography of Chinese characters. The orthographic forms of low-frequency characters would be less readily accessible via phonological activation than those of high-frequency characters, evoking greater activation in the inferior parietal lobule. This notion is in line with the observation of phonological activation in Chinese writing (Zhang and Damian, 2010; Qu et al., 2011; Damian and Qu, 2013). Another activation clusters in the parietal lobule was located in the bilateral superior parietal lobule (BA 7). Lesion studies (Sakurai et al., 2007) and neuroimaging studies (Purcell et al., 2011; Segal and Petrides, 2012; Planton et al., 2013) have repeatedly identified the superior parietal lobule as being engaged in writing processing. This region is considered as a key neural substrate for the motor components of writing, such as the storage of visuospatial or motor engrams for letters and words linked to handwriting (Sakurai et al., 2007). However, an electrical cortical stimulation study reported that stimulation of the anterior superior parietal lobule elicited both graphemic errors and lexical spelling errors in a writing-to-dictation task, suggesting that the superior parietal lobule is also involved in access to the spelling of lexical items (Magrassi et al., 2010). Thus, the increased activation in the superior parietal lobule for the low-frequency characters relative to high-frequency characters might result from the increased difficulty of accessing the complex visual configuration or motor representation of those Chinese characters. Additionally, the posterior parietal lobule has been found to be a crucial brain region for bottom-up attention to salient stimuli (Buschman and Miller, 2007). Consequently, the increased activation in the superior parietal lobule for low-frequency characters might be due to the bottom-up modulation of attention, because relative to low-frequency characters, high-frequency characters have an increased valence of familiarity.

### Functional connectivity associated with the word-frequency effect

Beyond the functional localization of isolated regions, the present study reveals the patterns of interregional communication underlying orthographic access for writing. First, PPI analysis demonstrated that the word-frequency effect in writing recruited neural connections within the fronto-occipital networks. Functional connectivity between the superior frontal gyrus (specifically, the SMA) and visual processing regions (the bilateral fusiform gyrus and the inferior/middle occipital gyrus) was sensitive to the frequency effect. The SMA has been found to be associated with the dynamic representation of the stroke sequence of Chinese characters (Yu et al., 2011). The left mid-fusiform gyrus has been suggested to subserve the storage of whole-word in the long-term memory (Dehaene et al., 2002; Dehaene and Cohen, 2011; Ludersdorfer et al., 2015), while the right fusiform gyrus is assumed to support the arrangement of the radicals of Chinese characters (Liu and Perfetti, 2003). Writing processing involves retrieving the whole-word orthographic representation and decomposing it into radicals or strokes, which relies on the connectivity between the SMA and the bilateral fusiform gyrus. Similarly, the bilateral middle occipital gyrus and the lingual gyrus have been proposed to be the dorsal route for visual-spatial processing in word recognition (Tan et al., 2001, 2005a; Cao et al., 2009; Xu et al., 2015). Xu et al. (2015) found that the bilateral inferior/middle occipital areas were sensitive to the number of strokes during reading of Chinese characters, suggesting that these occipital areas were involved in visual processing at the subcharacter level. Thus, the connection between the SMA and the middle occipital gyrus (lingual gyrus) might be recruited to integrate different levels of orthographic codes for written output.

Second, the functional connectivity between the left inferior frontal gyrus and the middle occipital gyrus (BA19) and left pre-/post- central gyrus was associated with the word-frequency effect. Given the role of the left inferior frontal gyrus in phonological processing (Poldrack et al., 1999; Tan et al., 2005a; Booth et al., 2007; Cone et al., 2008; Wu et al., 2012), the connectivity between the inferior frontal gyrus and visual-occipital cortex also suggests that phonological activation is necessarily coupled with visual-orthographic processing, supporting the hypothesis of obligatory phonological mediation in writing (Geschwind, 1969). On the other hand, the left inferior frontal gyrus has been reported to be involved in selection among orthographic forms during writing (Rapp and Dufor, 2011), while the middle occipital gyrus has been found to support the visual-spatial analysis of Chinese characters (Kuo et al., 2003; Li et al., 2004; Tan et al., 2005a). This functional connectivity between the left inferior frontal gyrus and the middle occipital gyrus could be conceived to represent the difference in resources demanded for selection of the proper orthographic configurations during written production. A prior study of effective connectivity has explained that backward connection from the middle frontal gyrus to the left vOT during Chinese reading (Xu et al., 2015) that was explained as a top-down control mechanism for improving low-level visual-orthographic processing (Cardin et al., 2010).

Finally, we found that the functional connectivity between the left inferior parietal lobule and the left lateral middle occipital gyrus (BA 17/18) was sensitive to the word-frequency effect. The left inferior parietal lobule is the neural substrate of the phonological storage of Chinese characters (Tan et al., 2005a). The dual-route model of visual word recognition posits that visual recognition of words involves two pathways, namely the ventral and dorsal pathways (Carreiras et al., 2014), and that connectivity between the middle occipital gyrus and the posterior parietal cortex is an important part of the dorsal pathway (Levy et al., 2009). Accordingly, we propose that the connectivity between the left inferior parietal lobule and the left lateral middle occipital gyrus is another phonological route for orthographic access in written production.

## Limitations

The following limitations must be considered in evaluating the present study and its outcomes. First, we did not observe the word-frequency effect in writing latency and motor execution, which seemingly was inconsistent with previous studies of Chinese writing (Zhang and Wang, 2014; Qu et al., 2016). One plausible explanation is the differences between the task paradigms. Written naming task used by Zhang and Wang (2014) and Qu et al. (2016) is more complex than the simple copying task used by the present study, which might be more sensitive to the word-frequency effect. Another possibility is that the number of participants is too small to reach the statistic threshold. Specifically, in Qu et al. (2016) study, the difference in writing latency between high-frequency and low-frequency characters was 36 ms, which is similar to the writing duration difference (40 ms) observed in the present study. Our correlation analysis revealed that writing latency and writing duration was significantly correlation. In this sense, we could argue that the lack of behavioral effect might not undermines our interpretation of the brain imaging results. Further studies recruiting more participants and applying more task paradigms are needed to certify the findings of the present study.

Second, writing in the scanner is different from the normal way of writing. For example, writing in the scanner would be slower than everyday writing due to the unfamiliar motions in the former task, although all the participants had undergone substantial practice before the MRI scan. Thus, one should consider to what extent our findings could apply to normal writing in daily life.

Finally, PPI analysis can hardly reveal the information flow between brain regions for orthographic access during writing. Further studies of effective connectivity are required to clarify the direction of these interactions between brain regions.

## Conclusion

In the present study, we used fMRI to unveil the brain activation and functional connectivity associated with the word-frequency effect during Chinese writing, involving several brain regions in the frontal, parietal, and occipital cortex. The findings illuminate the neural correlates of representing and accessing orthographic codes for written output in a logographic writing system, shedding new light on the cognitive processes of writing in various writing systems.

## Author contributions

YY, JZ and H-YB conceived and designed the experiment. YY, Z-LM, LQ and Y-FL performed the experiment. YY, JZ, Z-LM, LQ, and Y-FL performed the data analyses. YY, JZ, and H-YB co-wrote the paper. YY, JZ, Z-LM, LQ, and H-YB discussed the data and commented on the manuscript.

### Conflict of interest statement

The authors declare that the research was conducted in the absence of any commercial or financial relationships that could be construed as a potential conflict of interest.
