# Supplementary material for: Neural Correlates of Orthographic Access in Mandarin Chinese Writing: An fMRI Study of the Word-Frequency Effect
Source: Front Behav Neurosci. 2018 Nov 30;12:288. doi: 10.3389/fnbeh.2018.00288 (PMC6284029; doi:10.3389/fnbeh.2018.00288)
Supplement: Supplementary file 1 [file Table_1.DOCX]

Table 1 The attributes of the stimuli used in this study. HFC=high-frequency characters, LFC=low-frequency characters.

| HFC | Stroke | Frequency | LFC | Stroke | Frequency |
| --- | --- | --- | --- | --- | --- |
| 风 | 4 | 815 | 毋 | 4 | 1 |
| 电 | 5 | 1138 | 圩 | 6 | 0.5 |
| 次 | 6 | 982 | 伢 | 6 | 1 |
| 但 | 7 | 1302 | 轩 | 7 | 1 |
| 夜 | 8 | 514 | 昙 | 8 | 0.5 |
| 文 | 4 | 1175 | 讣 | 4 | 1 |
| 外 | 5 | 1364 | 叩 | 5 | 1 |
| 机 | 6 | 850 | 芊 | 6 | 1 |
| 块 | 7 | 518 | 岌 | 6 | 1 |
| 该 | 8 | 590 | 侠 | 8 | 1 |
| 化 | 4 | 745 | 刈 | 4 | 0.5 |
| 业 | 5 | 1217 | 汀 | 5 | 1 |
| 先 | 6 | 624 | 扪 | 6 | 1 |
| 花 | 7 | 871 | 芭 | 7 | 1 |
| 命 | 8 | 1545 | 沽 | 8 | 1 |
